# The Role of TRIP6, ABCC3 and CPS1 Expression in Resistance of Ovarian Cancer to Taxanes

**DOI:** 10.3390/ijms23010073

**Published:** 2021-12-22

**Authors:** Karolina Seborova, Alzbeta Kloudova-Spalenkova, Kamila Koucka, Petr Holy, Marie Ehrlichova, Changwei Wang, Iwao Ojima, Iveta Voleska, Petr Daniel, Kamila Balusikova, Michael Jelinek, Jan Kovar, Lukas Rob, Martin Hruda, Marcela Mrhalova, Pavel Soucek, Radka Vaclavikova

**Affiliations:** 1 Toxicogenomics Unit, National Institute of Public Health, 100 00 Prague, Czech Republic; karolina.seborova@szu.cz (K.S.); alzbeta.spalenkova@szu.cz (A.K.-S.); kamila.koucka@szu.cz (K.K.); petr.holy@szu.cz (P.H.); marie.ehrlichova@szu.cz (M.E.); voleskaiv@natur.cuni.cz (I.V.); pavel.soucek@lfp.cuni.cz (P.S.); 2 Laboratory of Pharmacogenomics, Biomedical Center, Faculty of Medicine, Charles University, 323 00 Pilsen, Czech Republic; 3 Third Faculty of Medicine, Charles University, 100 00 Prague, Czech Republic; 4 Institute of Chemical Biology & Drug Discovery, Stony Brook University—State University of New York, Stony Brook, NY 11794, USA; wachangw@gmail.com (C.W.); iwao.ojima@stonybrook.edu (I.O.); 5 Division of Cell and Molecular Biology, Third Faculty of Medicine, Charles University, 100 00 Prague, Czech Republic; petr.daniel@lf3.cuni.cz (P.D.); kamila.balusikova@lf3.cuni.cz (K.B.); michael.j@email.cz (M.J.); jan.kovar@lf3.cuni.cz (J.K.); 6 Department of Gynecology and Obstetrics, Third Faculty of Medicine and University Hospital Kralovske Vinohrady, 100 00 Prague, Czech Republic; lukas.rob@fnkv.cz (L.R.); martin.hruda@fnkv.cz (M.H.); 7 Department of Pathology and Molecular Medicine, Second Faculty of Medicine and Motol University Hospital, 150 06 Prague, Czech Republic; marcela.mrhalova@lfmotol.cuni.cz

**Keywords:** ovarian carcinoma, multidrug resistance, taxanes, Stony Brook taxanes, TRIP6, CPS1, ABCC3

## Abstract

The main problem precluding successful therapy with conventional taxanes is de novo or acquired resistance to taxanes. Therefore, novel experimental taxane derivatives (Stony Brook taxanes; SB-Ts) are synthesized and tested as potential drugs against resistant solid tumors. Recently, we reported alterations in *ABCC3*, *CPS1*, and *TRIP6* gene expression in a breast cancer cell line resistant to paclitaxel. The present study aimed to investigate gene expression changes of these three candidate molecules in the highly resistant ovarian carcinoma cells in vitro and corresponding in vivo models treated with paclitaxel and new experimental Stony Brook taxanes of the third generation (SB-T-121605 and SB-T-121606). We also addressed their prognostic meaning in ovarian carcinoma patients treated with taxanes. We estimated and observed changes in mRNA and protein profiles of ABCC3, CPS1, and TRIP6 in resistant and sensitive ovarian cancer cells and after the treatment of resistant ovarian cancer models with paclitaxel and Stony Brook taxanes in vitro and in vivo. Combining Stony Brook taxanes with paclitaxel caused downregulation of CPS1 in the paclitaxel-resistant mouse xenograft tumor model in vivo. Moreover, CPS1 overexpression seems to play a role of a prognostic biomarker of epithelial ovarian carcinoma patients’ poor survival. ABCC3 was overexpressed in EOC tumors, but after the treatment with taxanes, its up-regulation disappeared. Based on our results, we can suggest ABCC3 and CPS1 for further investigations as potential therapeutic targets in human cancers.

## 1. Introduction

Ovarian cancer is the seventh most common cancer in women worldwide, with around 240,000 new cases per year [1]. Most of these are epithelial ovarian carcinomas (EOCs) with the main aggressive histological subtype, the high-grade serous ovarian carcinoma (HGSC), accounting for 70% to 80% of all EOCs [2,3]. The high mortality of EOC is due to the absence of warning symptoms, biomarkers in body liquids, and specific screening procedures for detecting EOC in its early stages. The lack of these factors contributes to the suboptimal management of EOC. About 75–80% of cases are diagnosed at an advanced stage and have therefore poor prognosis, with a five-year survival rate of only 30% [4,5,6]. Similar to many other types of cancer, intrinsic or acquired multidrug resistance (MDR) to chemotherapy at advanced stages of EOC is the main problem preventing successful therapy [7,8]. 

The present standard therapeutic management of EOC consists of platinum-based chemotherapy, usually in combination with taxanes [9,10]. Resistance to conventional taxanes was recently summarized by Das et al. 2021, demonstrating the roles of alterations in microtubule or microtubule-associated proteins, alterations in the expression and activity of multidrug efflux transporters of the ATP binding cassette (ABC) superfamily including P-glycoprotein (P-gp/ABCB1), overexpression of anti-apoptotic proteins, or inhibition of apoptotic proteins and tumor-suppressor proteins as well as modulation of signal transduction pathways associated with the activity of several cytokines, chemokines, and transcription factors [8]. However, none of these potential biomarkers has been translated into clinical setting so far.

Resistance of EOC tumors to conventional anticancer therapies remains a serious problem and therefore new drugs and regimens to treat resistant tumors are sought. Recently, new therapeutic approaches have been introduced to the therapy of ovarian cancer, e.g., poly(ADP-ribose) polymerase inhibitors (PARPi), such as olaparib, or antiangiogenic agents such as bevacizumab or pazopanib [11,12]. These agents showed promising results in clinical trials. These novel therapeutic agents are tested in several clinical trials focused mainly on recurrent ovarian carcinoma patients with complete/partial response to the front line chemotherapy as a maintenance therapy [13]. However, even promising PARPi have limited efficacy in treatment of EOC patients with poor response to the front line chemotherapy and in platinum/paclitaxel resistant EOC patients [14]. Patients resistant to these regimens often do not regularly respond to PARPi as well. There is a significant overlap between mechanisms of resistance to platinum chemotherapy, and PARPi, with DDR alterations playing a key role. It is not yet clear whether patients who progress on PARPi, then respond to platinum chemotherapy, may retain some sensitivity to PARPi and benefit from second maintenance therapy with PARPi [15]. Another limitation of these novel drugs is their availability for patients and the price for the health system, especially in lower-income countries. An ongoing clinical trial focusing on the combination of PARPi and other targeted drugs such as the as Wee1 inhibitor (Adavosertib) seems to provide promising results for patients with progressive disease after front line chemotherapy [16]. 

**Figure 1:**
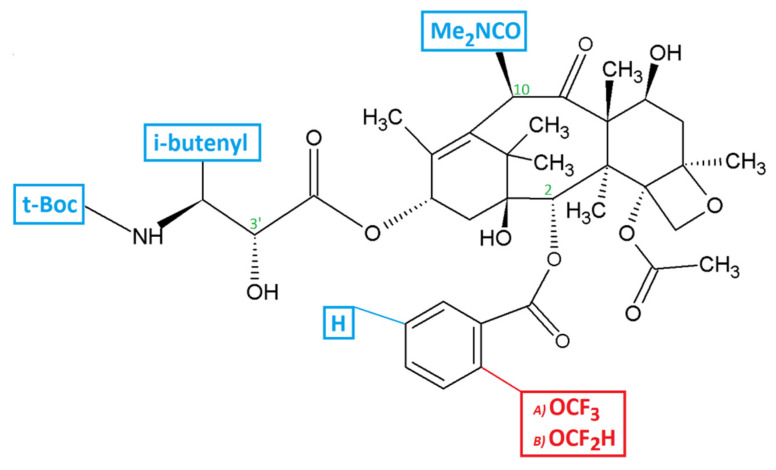
Structure formula of the novel taxane derivatives SB-T-121605 and SB-T-121606. Structures that differ from paclitaxel but are identical for new taxane derivatives are in blue. The different functional group between the two substances is in red—(A) SB-T-121605 and (B) SB-T-121606. Positions with synthetic modifications are in green (C-2, C-10, C-3′).

Novel synthetic taxane derivatives have been synthesized, e.g., Stony Brook Taxanes (SB-Ts) with synthetic modifications at the C-2, C-10, and C-3′ positions of paclitaxel (Figure 1) [17,18]. They seem to be highly effective in overcoming the ABCB1-dependent resistance of cancer cells in vitro [19,20,21,22,23,24]. Furthermore, the effect of the third generation SB-Ts was comparable to paclitaxel in non-tumorigenic human BEAS-2B cell line [25]. Considering the lack of response to PARPi in platinum-resistant patients, novel taxanes analogs could be additional way to treat the patients, especially those resistant to the front line of treatment. Until now, there is no biomarker for predicting the response to the taxane treatment that is routinely used in clinical setting, this being another area which needs more attention.

Complete elucidation of tumor resistance mechanisms is also investigated in the frame of cell targets with potential use as therapeutic targets. Recently, proteomic analyses of a paclitaxel-resistant, ABCB1 overexpressing, cancer cell model led to the discovery of several novel suspect molecules, particularly ABCC3 (ATP-binding-cassette subfamily C member 3), CPS1 (carbamoyl phosphate synthetase 1), and TRIP6 (thyroid hormone receptor-interacting protein 6) [22,26,27].

As regards the ABCC3 membrane transporter, its expression was documented to be significantly deregulated in different type of solid tumors. *ABCC3* was found to be increased in the histological HGSC subtype of EOC patients [28], as well as in cell line model of paclitaxel resistance in ovarian cancer (A2780/PTX) [29]. In our previous studies focused on the whole ABC transporter family expression in EOC patients [30,31], *ABCC3* transcript expression was found to be associated with shorter progression free survival after adjuvant chemotherapy based on paclitaxel and platinum derivatives combination [31]. In the other solid tumors, ABCC3 overexpression induced a resistant phenotype for methotrexate and doxorubicin in breast cancer cells [32] and it played the role in acquired resistance in HER2-amplified breast cancer [33]. Overexpression of ABCC3 was also found in resistant group of NSCLC (Non-Small Cell Lung Cancer) patients (treated by paclitaxel) compared to sensitive ones [34]. Furthermore, genetic variation identified in *ABCC3* gene (SNP rs1051640) was found to be associated with better progression-free survival in NSCLC patients treated with paclitaxel [35]. Very recently, Ramírez-Cosmes et al. summarized the implications of ABCC3 in cancer drug resistance [36].

Mitochondria play an essential role in apoptosis regulation, and they are also essential for cell metabolism and respiration, and cell signaling [37,38,39,40]. One of the mitochondrial proteins, a urea cycle enzyme carbamoyl-phosphate synthetase I (CPS1), is significantly overexpressed in breast cancer-resistant cell lines due to the increase in the number of CPS1 positive breast cancer paclitaxel-resistant cells as found by us [41]. The association of CPS1 deregulation with cancer therapy response is not known yet. One study has shown that an overexpression of CPS1 associated with poor chemo-radiotherapy response in rectal cancer [42].

Regarding the third candidate molecule, it was reported that TRIP6, a zyxin family member being enriched at focal adhesions [43], has been markedly upregulated in paclitaxel-resistant breast cancer MCF-7/PacR cells [27]. Notably, TRIP6 silencing decreased the number of viable MCF-7/PacR even expressing the functional ABCB1 transporter, making TRIP6 an attractive candidate molecule for further studies. How TRIP6 regulates cell proliferation or cell death in resistant cells has not been shown [27]. Until now, it was found that disruption of the functional complex of TRIP6 with LPA2, Siva-1, and TRIP6 knockdown attenuates LPA2 mediated protection from doxorubicin induced apoptosis [44] and also for cisplatin [45]. In general, significant deregulations of the mentioned candidate molecules, especially in resistant cancer cells, support their potential as therapeutic targets. Nevertheless, it is not known whether deregulation of ABCC3, CPS1 and TRIP6 occurs in different types of paclitaxel-resistant ovarian carcinoma cells or how the deregulation is affected by the action of paclitaxel and novel taxane derivatives.

Herein, we aimed to investigate the expression profile of the suspect molecules ABCC3, CPS1, and TRIP6 in ovarian carcinoma cell lines resistant to paclitaxel and reveal potential deregulation of the expression of ABCC3, CPS1 and/or TRIP6 after the treatment with paclitaxel and Stony Brook Taxane derivatives (SB-T-121605 and SB-T-121606) [18] in a model of ovarian carcinoma cells in vitro, and corresponding mouse tumor xenografts in vivo. The next goal of this study was to assess whether ABCC3, CPS1, and TRIP6 might serve as biomarkers of prognosis, therapeutic response, and survival of ovarian carcinoma patients for improving therapy personalization.

## 2. Results

### 2.1. mRNA and Protein Expression Profile of ABCC3, CPS1, and TRIP6 in Sensitive and Resistant Ovarian Carcinoma Cell Lines

We compared levels of ABCC3, CPS1, and TRIP6 mRNA and protein expression in various paclitaxel-resistant ovarian carcinoma cell lines; NCI/ADR-RES cell line cross-resistant to paclitaxel, and subclones of SKOV-3 and OVCAR-3 cells (named SKOV-3/RES and OVCAR-3/RES, respectively) with acquired resistance to paclitaxel. Furthermore, the levels of the examined genes were compared with sensitive SKOV-3 ovarian carcinoma cell line.

**Figure 2:**
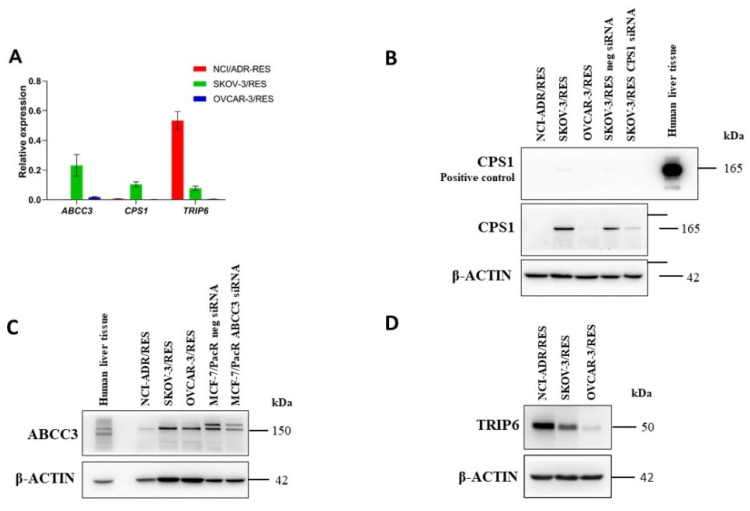
ABCC3, CPS1, and TRIP6 expression in paclitaxel-resistant NCI/ADR-RES, SKOV-3/RES, and OVCAR-3/RES. (**A**) Bar graph showing relative expression of *ABCC3, CPS1* and *TRIP6* genes in paclitaxel-resistant ovarian cancer cell lines (technical triplicates). (**B**) Representative immunoblots of CPS1 in paclitaxel-resistant ovarian carcinoma cell lines. CPS1 silenced SKOV-3/RES cells or non-specific siRNA transfected SKOV-3/RES cells and human liver tissue were used as controls. (**C**) Representative immunoblot of ABCC3 in paclitaxel-resistant cell lines. ABCC3 silenced MCF-7/RES breast cancer cells or non-specific siRNA transfected MCF-7/PacR breast cancer cells and human liver tissue were used as controls. (**D**) Representative immunoblot of TRIP6 in paclitaxel-resistant ovarian carcinoma cell lines. β-ACTIN served as a loading control. The size of TRIP6 band was confirmed previously by us [25].

In NCI/ADR-RES ovarian carcinoma cell line, we observed the highest level of *TRIP6* mRNA followed by *CPS1* and *ABCC3* mRNA (Figure 2A). In SKOV-3/RES cell subline, the highest level of *ABCC3* mRNA, followed by *CPS1* and *TRIP6* mRNAs was found. In the OVCAR-3/RES cell subline, the levels of all examined genes were poorly expressed in the order: *ABCC3* > *TRIP6* > *CPS1* as shown in Figure 2A. Protein expression of TRIP6 and CPS1 followed the same trend as observed at mRNA levels of those genes (Figure 2B,D). ABCC3 protein expression was the highest in SKOV-3/RES cell line, followed by low basal expression in OVCAR-3/RES and NCI/ADR-RES cell lines (Figure 2C), as observed also on mRNA levels. 

**Figure 3:**
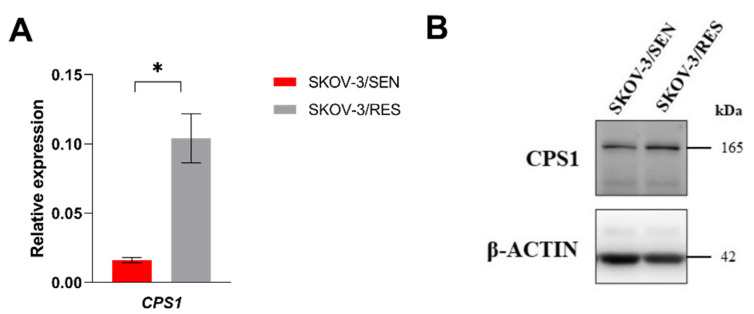
mRNA and protein levels of CPS1 in SKOV-3 ovarian carcinoma cell line and its paclitaxel-resistant subclone SKOV-3/RES in vitro. (**A**) Relative *CPS1* mRNA expression in SKOV-3/SEN and SKOV-3/RES was measured in technical triplicates. (**B**) Representative immunoblot of CPS1 protein expression in SKOV-3/SEN and SKOV-3/RES cell line. * *p*-value by two-tailed Student´s *t*-test (*p* < 0.05).

Subsequently, we performed comparison of ABCC3, CPS1, and TRIP6 expression in resistant ovarian SKOV-3/RES cell line and corresponding sensitive SKOV-3 cell line. We found a significant overexpression of *CPS1* gene (*p* = 0.02) (Figure 3A) also visible at the protein levels (Figure 3B), in the resistant SKOV-3/RES subclone in comparison to its parental sensitive SKOV-3 cell line.

NCI/ADR-RES cell line was selected for subsequent studies due to the *ABCC3, CPS1* and *TRIP6* genes having similar expression pattern when compared to EOC tumor samples described below. 

### 2.2. Effect of Paclitaxel and Novel Stony Brook Taxanes on ABCC3, CPS1, and TRIP6 Expression In Vitro

We measured the mRNA expression level of *ABCC3*, *CPS1*, and *TRIP6* in NCI/ADR-RES ovarian carcinoma cell line after 48 h cultivation with paclitaxel (3000 nM concentration), or novel generation taxanes SB-T-121605 and SB-T-121606 (300 nM concentration). The doses of paclitaxel and new generation SB-Ts have been selected on the basis of the highest induction of G2/M block estimated in the NCI/ADR-RES cell line in a study of their effect on cell cycle in our previous papers [20,21,46]. 

**Figure 4:**
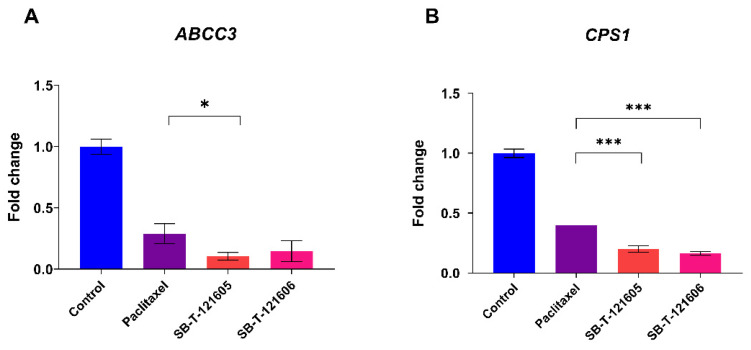
Significant differences in the expression of (**A**) *ABCC3* and (**B**) *CPS1* genes in NCI/ADR-RES cell line after the treatment with paclitaxel and novel Stony Brook taxanes, SB-T-121605 and SB-T-121606 in vitro. Difference in gene expression is displayed as mean of fold-change with ± SD (2^−∆∆CT^). Statistical analysis was performed by the two-tailed Student’s *t*-test (* *p* < 0.05, *** *p* < 0.001). Expression was measured in technical triplicates.

As shown in Figure 4, treatment with taxanes led to the significantly decreased mRNA level of *ABCC3* and *CPS1* genes. The mRNA level of the *TRIP6* gene was unchanged after the treatment with taxanes in the NCI/ADR-RES ovarian carcinoma cell line (data not shown). The decrease in *ABCC3* mRNA level after the treatment with SB-Ts was approximately twofold greater than after paclitaxel treatment, as shown by fold-change analysis in Figure 4A. In the case of the *CPS1* gene, fold-change estimation showed a significant decrease of *CPS1* mRNA levels after the treatment with paclitaxel (*p* < 0.001), SB-T-121605 (*p* < 0.001), and SB-T-121606 (*p* < 0.001, Figure 4B) in NCI/ADR-RES cell line. When we compared paclitaxel and SB-Ts treatments, we found significantly higher downregulation of *CPS1* after the treatment with novel SB-Ts for both SB-T-121605 (*p* < 0.001) and SB-T-121606 (*p* < 0.001) (Figure 4B).

### 2.3. Modulation of ABCC3, CPS1, and TRIP6 Expression by Novel Stony Brook Taxanes In Vivo

We then measured changes in the expression of *ABCC3, CPS1*, and *TRIP6* genes in a mouse xenograft model treated with paclitaxel alone or in separate combinations with the novel taxanes SB-T-121605 and SB-T-121606 in vivo. At first, the toxicity of taxanes during the in vivo i. p. application was tested. Experimental mice were treated solely with SB-Ts or their combinations with paclitaxel, and DMSO as a vehicle. The application of DMSO alone was not toxic for experimental mice below a 5% concentration. SB-Ts alone were tested in range of concentration doses from 1 mg/kg to 8 mg/kg. The toxic effects of SB-Ts were observed in concentrations higher than 3 mg/kg, so for the main experiment we used combinations based on lower doses of SB-Ts compared to paclitaxel. The toxicity was presented by bowel obstruction and the overall physical wasting of mice. Therefore, combinations of SB-Ts with paclitaxel were investigated by this study as a potentially efficient and less toxic regimen with preservation of the SB-Ts effect on tumor growth. Combinations of SB-Ts with paclitaxel were tested as follows; 1 and 3 mg/kg of SB-T with 9 and/or 7 mg/kg of paclitaxel. Experimental mice models have not suffered with life threatening toxicity (no deaths have been observed during the two-week experiment duration and application of three doses) which demonstrates the good toxicity profiles of all drug regimens used. Maximum single dose of taxanes (alone or combined) in all experiments was set to 10 mg/kg, which is well tolerated by mice when applied twice a week in contrast to a single dose of 20 mg/kg once a week as we observed for paclitaxel (data not shown).

**Figure 5:**
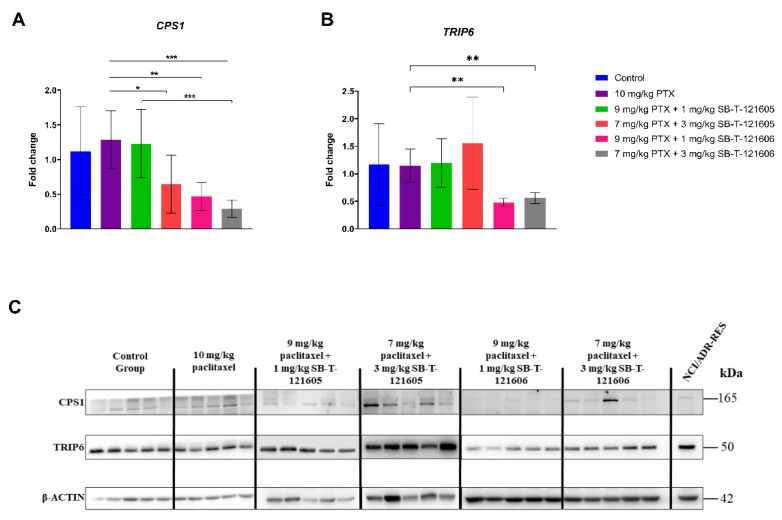
Significant differences in the mRNA levels of (**A**) *CPS1* and (**B**) *TRIP6* genes and (**C**) CPS1 and TRIP6 proteins in ovarian carcinoma mouse xenografts after the treatment with paclitaxel and novel SB-Ts in vivo. (**A**,**B**) Gene expression differences are shown as a mean of fold change (2^−∆∆CT^) ± SD, between the control group (Group I), group treated with 10 mg/kg paclitaxel (Group II), 9 mg/kg paclitaxel + 1 mg/kg SB-T-121605 (Group III), 7 mg/kg paclitaxel + 3 mg/kg SB-T-121605 (Group IV), 9 mg/kg paclitaxel + 1 mg/kg SB-T-121606 (Group V), and 7 mg/kg paclitaxel + 3 mg/kg SB-T-121606 (Group VI). Statistical analysis was performed by the two-tailed Student´s *t*-test * *p* < 0.05, ** *p* < 0.01, *** *p* < 0.001). (**C**) Representative immunoblot of CPS1, TRIP6, and β-ACTIN proteins in each group of mouse xenografts. Each group consisted of five mice.

In our in vivo experimental part of the study, tumor xenograft models of resistant ovarian cancer were prepared from the NCI/ADR-RES cell line, and each experimental group consisted of five mice. When compared to the control group I, we did not find any significant changes of the examined mRNA levels in the paclitaxel group (Group II). On the other hand, we found a significant decrease in the expression of the *CPS1* gene after the treatment with novel taxanes in combination with paclitaxel. Particularly, significant downregulation of the *CPS1* gene was found in ovarian tumors after the treatment with combinations of 9 mg/kg paclitaxel with 1 mg/kg SB-T-121606 (Group V; *p* = 0.004) and 7 mg/kg paclitaxel with 3 mg/kg SB-T-121606 (Group VI; *p* < 0.001) compared to paclitaxel alone (Group II, Figure 5A). Expression of *CPS1* was also downregulated by 7 mg/kg paclitaxel with 3 mg/kg SB-T-121605 combination (Group IV) compared to paclitaxel alone (Group II; *p* = 0.042, Figure 5A). Downregulation of the *CPS1* gene after the treatment with taxanes in vivo was in concordance with results observed in NCI/ADR-RES cells treated with taxanes in vitro (Figure 4B). Furthermore, we found significant changes in *TRIP6* mRNA level after the treatment with SB-Ts. Particularly, the treatment of mice with combinations of 9 mg/kg paclitaxel with 1 mg/kg SB-T-1621606 (Group V, *p* = 0.001) and 7 mg/kg paclitaxel with 3 mg/kg SB-T-121606 (Group VI, *p* = 0.003) led to a significant decrease in the mRNA level of *TRIP6* gene in comparison to the group treated with paclitaxel alone (Group II) (Figure 5B). In contrast to in vitro experiments, the downregulation of *ABCC3* mRNA level was not found in vivo after the treatment of mice with taxanes (data not shown). However, the level of *ABCC3* expression in vivo was very low in general. To confirm the significant results found at the mRNA level, we measured the levels of CPS1 and TRIP6 proteins in all groups of the examined xenografts. The significant decrease of CPS1 and TRIP6 expression was also detected at protein levels for groups V and VI of combination regimens of paclitaxel and SB-T-121606 in comparison to the group treated with paclitaxel alone (Figure 5C). mRNA and protein levels of CPS1 were correlated in Group III (*p* = 0.037) and Group IV (*p* = 0.037) by the Spearman´s rho test.

### 2.4. EOC Study Population

#### 2.4.1. Patients Characteristics

We further examined the expression profile of ABCC3, CPS1, and TRIP6 directly in the cohort of EOC patients. Clinical data, response to the therapy, and survival of patients who provided tissue samples of EOC tumors (*n* = 113) are in Table 1. Samples from 89 EOC patients were collected during primary surgery without any prior chemotherapy pretreatment (Pretreatment Group). Samples of the second group of patients (*n* = 24) were collected during surgery after neoadjuvant cytotoxic therapy (NACT) using regimens containing paclitaxel in combination with platinum derivatives (Posttreatment Group) as described in detail in Table 1. The median age (±SD) at the time of diagnosis of patients with EOC was 59.8 ± 10.8 years. Most of the EOC patients had High Grade Serous Ovarian Carcinomas (HGSC; 79.6%), grade 3 tumors (77.0%), and were at advanced stages III and IV (81.4%). In order to determine therapy response, we divided all tumor samples based on the platinum-free interval (PFI), defined as the interval between the date of the last platinum dose and the date of relapse detection [47,48]. EOC patients were divided into platinum-resistant (*n* = 23; PFI length < six months), partially platinum-sensitive (*n* = 15; PFI length from six to 12 months), and fully platinum-sensitive (*n* = 70; PFI length > 12 months). Disease progression occurred in 69 of 113 EOC patients and 43 EOC patients died. The median time to progression (TTP) (± SD) of EOC patients included in the study was 22 months. Tissue samples of 17 patients without morphological signs of primary ovarian carcinoma in their ovaries (ovarian leiomyoma, *n* = 6; uterine leiomyoma, *n* = 1; benign ovarian cyst, *n* = 4; cervical carcinoma, *n* = 2; endometrial carcinoma, *n* = 2; sarcoma, *n* = 1; benign cystadenofibroma, *n* = 1) were used as controls.

#### 2.4.2. ABCC3, CPS1, and TRIP6 Expression Profile in EOC Patients

We measured the mRNA level of *ABCC3, CPS1,* and *TRIP6* in the cohorts of EOC patients (*n* = 113) and control ovarian tissues without the presence of malignant cells (*n* = 17). Level of mRNA of all genes was successfully detected in EOC tumors and control ovarian tissues. In concordance with results observed in the in vitro model of paclitaxel-resistant ovarian carcinoma cell line NCI/ADR-RES, we observed the highest level to be that of *TRIP6* mRNA, followed by *ABCC3* and *CPS1* transcripts in our set of EOC tumors. In EOC patients, the mRNA levels of the three genes correlated highly significantly with each other (the Spearman´s rho test; *p* < 0.001). Subsequently, we compared the mRNA level of *ABCC3, CPS1,* and *TRIP6* genes in EOC tumor samples with control ovarian tissues. The mRNA levels of *TRIP6* and *CPS1* were significantly decreased in EOC pretreatment and also posttreatment tumors in comparison to control ovarian tissue (Table 2). The mRNA level of the *ABCC3* gene was elevated in tumor samples before the chemotherapeutic treatment, while this effect disappeared after the treatment (Table 2). The same trend was observed in the in vitro model of ovarian carcinoma cell lines, where the treatments with taxanes caused downregulation of the *ABCC3* expression. 

**Figure 6:**
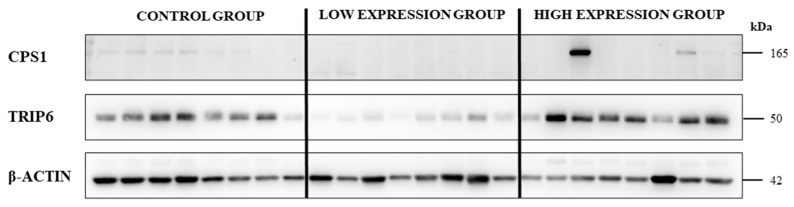
Protein levels of CPS1 and TRIP6 in control ovarian tissues and EOC patients divided according to their mRNA expression to low and high expression groups. Protein levels were estimated in tumor and control ovarian tissues by immunoblotting. Each group consisted of eight randomly selected samples.

Subsequently, we compared the expression of mRNA levels of *CPS1* and *TRIP6* with their protein levels in representative sets of control ovarian tissues and EOC tumor samples divided into EOC low and high mRNA expression groups (Figure 6). As shown on Figure 6, the protein levels of TRIP6 and CPS1 reflect low and high expression of mRNA. Nevertheless, the expression of CPS1 and TRIP6 mRNA and protein levels did not correlate significantly (the Spearman´s rho test; *p* = 0.528 and 0.260, respectively). On the other hand, downregulation of CPS1 and TRIP6 protein in the low mRNA expression group was highly significant (Student´s *t*-test; *p* < 0.01) in comparison to control ovarian tissues. TRIP6 protein expression was also significantly higher in the high mRNA expression group compared to the low expression group of EOC patients (Student´s *t*-test; *p* < 0.01), as shown in Figure 6. 

#### 2.4.3. Association of ABCC3, CPS1, and TRIP6 Gene Expression with Clinical Data

**Figure 7:**
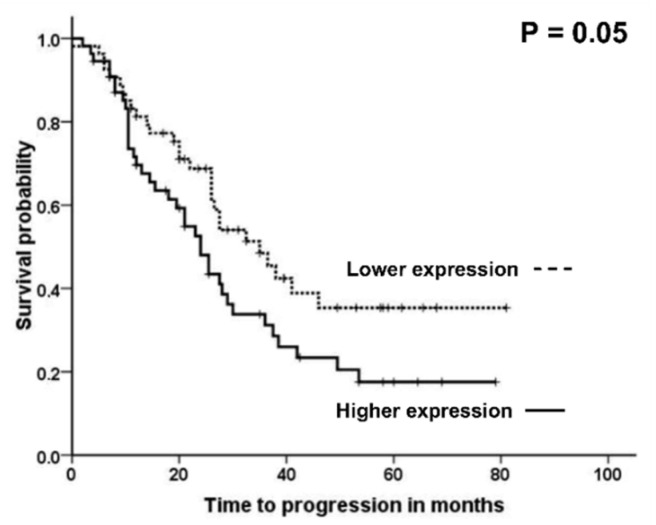
Association between expression level of *CPS1* gene and time to progression of EOC patients. Survival curves for patients with the intratumoral *CPS1* expression levels above the median (solid line, *n* = 55) vs. patients with lower expression than the median (dashed line, *n* = 54) are displayed. Results evaluated by the Kaplan-Meier plot. Significance was evaluated by the log-rank test.

Finally, we compared the expression of *ABCC3, CPS1,* and *TRIP6* genes with the clinical data of EOC patients, such as grade, stage, histology type, progression of the disease, therapeutic response, and survival estimated as TTP. There was no association between mRNA expression of *ABCC3*, *CPS1*, and *TRIP6* and pathological data, the prognosis of EOC, progression, or the therapeutic response estimated based on PFI. On the other hand, we found a suggestive association of *CPS1* mRNA expression with TTP of EOC patients. Patients with higher than median intra-tumoral *CPS1* gene expression had significantly shorter TTP than the rest of the patients (Figure 7; the log rank test; *p* = 0.05). Survival analysis was performed by the Kaplan-Meier method, and the log-rank test was applied to identify significant associations.

## 3. Discussion

The main problems in the successful treatment of ovarian carcinoma are late diagnosis of this disease and the MDR phenomenon. At present, new therapeutics and molecular targets in the therapy of resistant ovarian tumors are needed together with biomarkers indicating which drugs offer the highest chance for successful treatment. In our previous publications, we reported several new molecules potentially involved in the resistance of cancer cells to taxanes, such as ABCC3, CPS1, and TRIP6 [22,26,27,41]. In the present study, we investigated the expression profile of these candidate molecules in sensitive and different models of resistant ovarian carcinoma cell lines and potential dysregulation caused by the treatment with a conventional taxane, paclitaxel, and synthetic derivatives Stony Brook taxanes in vitro and in vivo. Furthermore, we also explored the impact of ABCC3, CPS1, and TRIP6 mRNA and protein levels on prognosis and the therapeutic outcome in EOC patients. 

Dysregulations of the expression of ABC membrane transporters, e.g., ABCB1, ABCC1, ABCC2, and ABCG2, can be associated with the development of MDR [49,50,51], but ABCC3 (alias MRP3; member of ABCC subfamily), active in the transport of conjugated organic anions, toxicants, drugs, and endogenous compounds, remains less explored. ABCC3 is associated with the sensitivity to anticancer drugs such as methotrexate or docetaxel and the selective estrogen receptor modulator tamoxifen (as summarized in [52]). ABCC3 is also involved in glutathione transport in ovarian cancer cells [49]. We described previously that it is in overexpressed subclones of breast cancer cell lines MCF-7 and SK-BR-3 with acquired resistance to paclitaxel when comparing with parental paclitaxel-sensitive MCF-7 and SK-BR-3 clones [22]. Recent reports demonstrated that disruption of ABCC3 function reduces pancreatic cancer cell growth in vitro and in vivo [53,54]. In ovarian cancer, ABCC3 protein is overexpressed in paclitaxel-resistant A2780/PTX cell line in vitro [29] and upregulated on the transcript level in histological HGSC subtype of EOC patients [28]. In the present study, we found upregulation of ABCC3 in EOC tumors compared to benign ovarian tissues. This observation is in concordance with the previous study, which described significantly elevated *ABCC3* gene expression in recurrent cancer lesions compared to benign ovarian tissue [55]. Moreover, we observed that the *ABCC3* level was decreased after neoadjuvant chemotherapy of EOC patients with a regimen combining taxanes and platinum derivatives. We observed the same effect here also in the highly paclitaxel-resistant ovarian cancer cells (NCI/ADR-RES) in vitro after the treatment with paclitaxel or the new synthetic Stony Brook taxanes, which are highly effective in the resistant type of tumor cells [18,19,20,21,24,51]. The strong decrease of ABCC3 expression after the treatment with taxanes suggests that ABCC3 may play a role in taxane transport. Thus, ABCC3 seems to be a novel and promising therapeutic target for ovarian carcinomas, where taxanes are usually used. Congruently, epigenetic regulation of ABCC3 expression by the overexpression of miRNA-200a in vitro enhanced the chemosensitivity of paclitaxel-resistant ovarian SKOV-3 and ES-2 cell lines to paclitaxel [56]. The *ABCC3* gene was also co-expressed with the non-coding RNA CTD-2589M5.4 [29]. In connection to novel therapeutic strategies in ovarian cancer, novel interactions between olaparib and ABCC3 were found very recently [57].

Our study revealed that CPS1 (carbamoyl phosphate synthetase 1) is expressed in the resistant ovarian carcinoma cell line model. CPS1 was significantly overexpressed in resistant SKOV-3 ovarian carcinoma cells in comparison to sensitive SKOV-3 cells and its higher gene expression level was also associated with worse survival rates of EOC patients. Treatment with taxanes led to downregulation of CPS1 in resistant in vitro and in vivo ovarian cancer models. In particular, the combining of Stony Brook taxanes with paclitaxel caused downregulation of CPS1 in the paclitaxel-resistant mouse xenograft tumor model in vivo in comparison to paclitaxel alone. CPS1 is a mitochondrial enzyme that significantly catalyzes the first step of the urea cycle. It was found to be upregulated in breast cancer MCF-7 cells with acquired resistance to paclitaxel when comparing with original sensitive MCF-7 cells [41]. Only a few studies demonstrated the potential association of CPS1 with tumor resistance [42,58], and the role of this mitochondrial protein in ovarian cancer remained completely unknown to date. Very recently, CPS1 downregulation of CPS1 expression in hepatocellular carcinomas and a further reduction in recurrent tumors and distant metastases was reported [59]. CPS1 knockdown stimulates soluble adenylyl cyclase expression, thereby increasing cyclic AMP (cAMP) synthesis and stimulating PKA-CREB/ATF1 signaling. Regulation of cAMP-PKA-CREB/ATF1 signaling represents a non-canonical function of CPS1, and targeting of the PKA-CREB/ATF1 axis may improve the therapeutic effects of aspirin in hepatocellular carcinoma [60]. Combining knockdown of CPS1 with EGFR inhibition reduces cell proliferation and impedes cell-cycle progression. Thus, suppression of CPS1 potentiates the effects of EGFR inhibition [61]. Among other regulators, CPS1 is activated in the presence of N-acetyl-L-glutamate (NAG) [62] and its transcription is negatively regulated by liver kinase B1 in lung adenocarcinoma cell lines [63,64]. Briefly, CPS1 functions in different ways depending on cell and tissue type and the presence of its activators. The present study observed for the first time a significantly decreased expression of CPS1 in the NCI/ADR-RES paclitaxel-resistant ovarian cancer cells in vitro and had the same effect also in the NCI/ADR-RES-xenografted mouse model in vivo after taxane treatment. Moreover, EOC patients with higher *CPS1* expression experienced a significantly shorter time to progression of their disease. Thus, our study shows that downregulation of the *CPS1* gene may be a putative prognostic biomarker in EOC patients. 

TRIP6 (thyroid hormone receptor interacting protein 6) is an adaptor protein involved in various types of signaling, including pro-survival and anti-apoptotic signaling. TRIP6 deregulations in various cancers may have pleiotropic roles in tumor initiation, tumor growth, and metastasis as summarized in Willier [65]. High expression of TRIP6 led to worse survival of non-Hodgkin´s Lymphoma (NHL) patients and it is associated with the accelerated proliferation of NHL cells [66]. Recent evidence supports TRIP6 engagement in Wnt signaling, indicating that TRIP6 might participate in regulation of cell proliferation [67]. We have recently shown that the TRIP6 protein is significantly upregulated in breast cancer MCF-7 cells with acquired resistance to paclitaxel compared to the original sensitive MCF-7 cells. Furthermore, specific siRNA silencing revealed that TRIP6 is involved, together with the ABCB1 transporter (P-glycoprotein), in the development of resistance of MCF-7 cells to paclitaxel [26,27]. In the present study we observed that TRIP6 is strongly overexpressed in a paclitaxel-resistant EOC model NCI/ADR-RES in vitro. Although the expression of the TRIP6 gene or protein was unchanged by taxane treatment of resistant ovarian cancer cell lines in vitro, the treatment of mice xenografts based on an NCI/ADR-RES model with experimental taxoid SB-T-121606 led to the TRIP6 protein downregulation in vivo. However, SB-T-121606 seems to cause downregulation of transcriptome in general by its extremely high efficacy towards resistant tumor cells. TRIP6 expression in tumor tissues from EOC patients was lower than that in control ovarian tissue and did not correlate with the survival of patients or their therapeutic outcome. In summary, our data show that TRIP6 cannot serve as a therapeutic target or prognostic biomarker in ovarian cancer at present. 

In conclusion, this study revealed the expression profile of three candidate molecules: ABCC3, CPS1, and TRIP6, previously associated with MDR phenomena, in resistant and sensitive ovarian carcinoma cell lines and EOC patients. CPS1 was significantly upregulated in the resistant type of ovarian cancer cells. After the treatment with conventional paclitaxel and synthetic Stony Brook taxanes, significant dysregulation of expression of candidate molecules in highly resistant ovarian carcinoma cell lines in vitro and also in their mouse xenograft in vivo version was found. Furthermore, significant dysregulation of ABCC3, CPS1, and TRIP6 expression in tumors from EOC patients was revealed. TRIP6 was not associated with the prognosis or survival of EOC patients, but high levels of CPS1 seem to be associated with worse survival rates of EOC patients. This finding is consistent with significantly higher levels of CPS1 expression revealed in resistant ovarian cancer cell lines in comparison to sensitive SKOV-3 cells. *ABCC3* was overexpressed in EOC tumors, but after the treatment with taxanes, its upregulation disappeared. Our findings provide new evidence that ABCC3 and CPS1 may act as mediators of therapy response in ovarian cancer cells. Future investigations should decipher molecular mechanisms of their function in cancer cells. 

## 4. Materials and Methods

### 4.1. Materials

Paclitaxel for in vitro experiments was obtained from Sigma Aldrich (St. Louis, MA, USA). Novel third generation taxane derivatives (SB-T-121605 and SB-T-121606) were synthetized at the Institute of Chemical Biology & Drug Discovery (Stony Brook, NY, USA). Chemical structures of the drugs examined are shown in Figure 1. All taxanes were dissolved in DMSO for stock and working solutions. Infusion form of paclitaxel (Paclitaxel EBEWE 6 mg/L) for in vivo experiment was purchased from Ebewe Pharma Ges.m.n.H.NfG.KG., Unterach am Attersee, Austria).

### 4.2. Cells and Culture Conditions

Human ovarian carcinoma cell lines sensitive to paclitaxel—OVCAR-3 and SKOV-3—were obtained from Cell Lines Service (CLS, Eppelheim, Germany). A model of multi-drug resistant ovarian carcinoma—NCI/ADR-RES cell line—was obtained from National Cancer Institute (Frederick, MD, USA). All cell lines were cultivated in RPMI 1640 medium (PAN-Biotech GmbH, Aidenbach, Germany) with L-glutamine (300 mg/L), NaHCO_3_ (2.0 g/L), penicillin (100 U/mL), streptomycin (100 μg/mL), sodium pyruvate (1 mM), HEPES (15 mM), and 10% fetal bovine serum (PAN-Biotech) at 37 °C in a humidified atmosphere with 5% CO_2_. Paclitaxel-resistant OVCAR-3/RES and SKOV-3/RES have been prepared by multistep selection procedure from OVCAR-3 and SKOV-3 cell lines cultivated in growth medium to final concentration of 300 nM (for OVCAR-3/RES), or 500 nM (for SKOV-3/RES) of paclitaxel. For expression analysis, cells were harvested as described in Section 4.3.

### 4.3. Cell Line Treatment with Paclitaxel and Novel Stony Brook Taxanes

NCI/ADR-RES cells were seeded in concentration 4 × 10^6^ cells into Petri dish and allowed to adhere overnight. After that, growth medium was replaced with fresh medium (control) or medium containing 3000 nM paclitaxel, 300 nM SB-T-121605 or 300 nM SB-T-161606. After 48 h of incubation, cells were harvested by trypsinization and low-speed centrifugation, washed with PBS twice. Pellets were resuspended in 1 mL of TRIzol^TM^ Reagent (Invitrogen^TM^, Waltham, MA, USA) and stored at −80 °C for later RNA isolation.

### 4.4. Xenografts

The study conducted on xenografts was approved by the Ministry of Agriculture of the Czech Republic and the Ethical Committee of the National Institute of Public Health in Prague. Female athymic Nude Crl:NU(NCr)-Foxn1^nu^ mice, four to six weeks old, were obtained from Velaz, s.r.o. (Prague, Czech Republic). NCI/ADR-RES cells were harvested, and the pellet was washed twice by PBS. The animals were injected subcutaneously into the dorsal flanks with 200 μL of the cell suspension containing 2 × 10^6^ cells in PBS. The treatment with taxanes was initiated after tumors reached the size of approximately 100 mm^3^.

### 4.5. In Vivo Treatment with Paclitaxel and Novel Stony Brook Taxanes

In total, 30 xenografts were prepared and divided into six groups: (I) Control group (*n* = 5) and experimental groups (*n* = 5 each) as follows: (II) 10 mg/kg paclitaxel, (III) 9 mg/kg paclitaxel + 1 mg/kg SB-T-121605, (IV) 7 mg/kg paclitaxel + 3 mg/kg SB-T-121605, (V) 9 mg/kg paclitaxel + 1 mg/kg SB-T-121606, and (VI) 7 mg/kg paclitaxel + 3 mg/kg SB-T-121606. These regimens were administered intraperitoneally twice a week, 100 μL per each taxane solution. Control group I received 100 μL of 4% DMSO in sterile water for tissue culture (PAN-Biotech) instead of taxanes. Mice were sacrificed on the day after the seventh dose or on the basis of their physical condition during taxane application. Tumor volume was measured by digital caliper in weekly intervals and expressed in mm^3^ using the standard formula, (W2 × L)/2, where L and W are the major and minor diameters of the tumor in millimeters. Resected tumors were preserved in RNA later (Sigma-Aldrich) and stored at −80 °C till further processing.

### 4.6. Patients Cohort Study

The present study tested ovarian carcinoma tissue samples obtained from 89 pretreatment and 24 posttreatment samples diagnosed with EOC at University Hospital Kralovske Vinohrady and Motol University Hospital (Prague, Czech Republic) during the period 2009–2016. Other 17 samples of ovarian tissues without morphological signs of carcinoma were used as controls in this study. Control samples were obtained from patients who underwent surgery for a different reason than ovarian malignancy. The tissue samples collected during surgery were histopathologically examined according to standard diagnostic procedures. The tissue samples were fresh-frozen and stored at −80 °C until isolation of RNA, DNA, and protein. The following data on patients were retrieved from medical records: the patients age at the time of diagnosis, FIGO stage, tumor grade, and type of EOC, expression of protein marker Ki67 in percentage points (available only for patients from Motol University Hospital), progression of disease, resistance to therapy (based on platinum derivatives), death, and time to progression (TTP) in months as specified in Table 1.

All patients were informed about the aims of the present study and provided their written consent to participate in the study. The design of the study was approved by the Ethics Commission of the National Institute of Public Health (Prague, Czech Republic), University Hospital Kralovske Vinohrady, and Motol University Hospital).

### 4.7. Isolation of Nucleic Acids and cDNA Synthesis

Tumor tissue samples from animals and ovarian cancer patients were homogenized by mortar and pestle under liquid nitrogen. Total RNA, together with DNA and protein, was isolated by AllPrep DNA/RNA/protein Mini kit (Qiagen, Hilden, Germany) according to the manufacturer´s protocol. Total RNA from cells was isolated by TRIzol^TM^ Reagent (Invitrogen^TM^) according to the manufacturer´s protocol. RNA quantity was determined by Quant-iT™ RiboGreen™ RNA Assay Kit (Invitrogen™) using Infinite M200 fluorescence reader (Tecan, Männedorf, Austria). Quality of RNA was assessed by estimating the RNA integrity number (RIN) using Agilent 2100 Bioanalyzer (Agilent Technologies, Santa Clara, CA, USA). The RIN value was 8.6 on average (range 7.7–9.6). RNA was totally degraded in one carcinoma tissue sample and therefore was not further evaluated. Complementary DNA (cDNA) was synthesized using 0.5 µg of total RNA by RevertAid First Strand cDNA Synthesis Kit (MBI Fermentas, Vilnius, Lithuania) according to the manufacturer´s protocol and its quality was confirmed by PCR amplification of Ubiquitin C fragment as described previously [68].

### 4.8. Quantitative Real-Time PCR

Quantitative real-time PCR (qPCR) was performed using TaqMan^®^ Gene Expression Assays (ThermoFisher, Waltham, MA, USA). TaqMan^®^ Gene Expression Assays selected for this study were CPS1 (Hs00919490_m1), TRIP6 (Hs00377979_m1), and ABCC3 (Hs000358656_m1). Highly stable expression of reference gene *YWHAZ* (Hs03044281_g1) was used for normalization of results in used in vitro and in vivo models. Genes *PPIA* (Hs99999904_m1), *UBC* (Hs00824723_m1), and *YWHAZ* (Hs03044281_g1) were used as reference genes for results normalization in ovarian cancer patients. The reaction mixture of cDNA from tumor samples contained 1 µL of 5× Hot FirePol Probe qPCR Mix Plus (ROX) (Solis BioDyne OÜ, Tartu, Estonia), 0.25 µL of 20× TaqMan Gene Expression Assay, 1.75 µL of nuclease-free water, and 2 µL of 8-times diluted cDNA to make a final reaction volume of 5 µL. PCR reaction was performed on 384-well position ViiA7 Real-Time PCR System (Life Technologies, Carlsbad, CA, USA). The reaction mixture of cDNA from treated and untreated cell line samples contained 5 µL of 2× Gene Expression Master Mix (ROX) (ThermoFisher), 0.5 µL of 20× TaqMan Gene Expression Assay, 2.5 µL of nuclease-free water, and 2 µL of 6-times diluted cDNA to make a final reaction volume of 10 µL. The PCR reaction was performed on 72-well position RG6000 system (Corbett Research, Mortlake, Australia). Cycling parameters of all reactions were initial hold at 50 °C for 2 min and 10 min denaturation at 95 °C followed by 45 cycles consisting of 15 s denaturation at 95 °C and 60 s annealing/extension at 60 °C. The non-template control (NTC) contained water instead of cDNA. Negative cDNA synthesis controls (RNA transcribed without reverse transcriptase) were also employed to reveal possible carry-over contamination. Samples were analyzed in duplicates; samples with a standard deviation of duplicates > 0.5 Ct were re-analyzed. Design of the qPCR study adhered to the MIQE guidelines [69].

### 4.9. Immunoblotting Analysis of Protein Expression

Western blot analyses were performed similarly as described previously [51]. Briefly, protein concentration in samples was determined using the Pierce BCA Protein Assay Kit (ThermoFisher). Samples were separated in hand casted 12% polyacrylamide gels and blotted onto a 0.2 µm nitrocellulose membrane for 3 h in Towbin buffer (25 mM Tris, 192 mM glycine, 20% methanol, pH 8.3). The membranes were blocked with 5% BSA in TBS buffer (100 mM Tris-HCl, 150 mM NaCl, pH 7.5). Following primary antibodies were applied onto the membranes and incubated overnight at 4 °C: *anti*-TRIP6 (HPA052813) and *anti*-ACTIN (clone AC-40) (A3853) from Merck (Darmstadt, Germany), *anti*-CPS1 [EPR7493-3] (ab129076) from Abcam (Cambridge, UK) and anti-MRP3 (PA5-23653) from ThermoFisher. Secondary HRP-conjugated antibodies, applied onto the washed membranes and incubated for 2 h at room temperature, were goat *anti*-mouse (SA00001-1) and goat *anti*-rabbit (SA00001-2) from Proteintech (Rosemont, IL, USA). Chemiluminescence signal was initiated using the enhanced SuperSignal™ West Pico PLUS Chemiluminescent Substrate (ThermoFisher) and images were taken using a CCD camera GEL Logic 4000 Pro (Carestream Health, Woodbridge, CT, USA). As a positive control of CPS1 and ABCC3 detection a human liver tissue protein sample was used. For a negative control of CPS1 and ABCC3 detection, samples created via knockdown of CPS1 and ABCC3 gene using Silencer^®^ Select siRNA ID s3462 and s16600, respectively, were used. Nonspecific Silencer^®^ Select siRNA 4390844 was used as a negative control of the procedure. All siRNAs were purchased from ThermoFisher. Cells were transfected via INTERFERin^®^ reagent (PolyPlus-Transfection, Illkirch, France) in Opti-MEM^®^ Reduced Serum Medium (ThermoFisher) according to manufacturer instructions and previously described in [41] with following modifications: The final concentration of CPS1 and ABCC3 siRNAs well as of corresponding negative controls was 5nM (CPS1) and 50nM (ABCC3) of siRNA in the culture medium. After 72 h of incubation with siRNA, cells were harvested and CPS1 and ABCC3 silencing was analyzed using western blot (see above). Original western blot images for Figure 2 and Figure 3 are listed in Figures S1 and S2 (Supplementary Materials).

### 4.10. Statistical Analyses

In vitro and in vivo estimated gene expression differences were calculated from raw Ct values as the fold change due to treatment in accordance with the comparative Ct method described by Livak and Schmittgen (2001). The 2^−ΔCt^ method was used for relative quantification, and the 2^−ΔΔCt^ method was used for fold change (FC) estimation in groups divided by the treatment with taxanes [70,71].

Statistical comparison between treated and untreated tumor cells and xenograft groups was performed by the two-tailed Student´s *t*-test in GraphPad Prism v4.0 software (GraphPad Software, San Diego, CA, USA). Protein levels were analyzed using densitometry performed in the Image MasterTM 2D Platinum 6.0 software (GE Healthcare, Uppsala, Sweden). The transcript levels of target genes were normalized to reference genes listed in chapter 4.8 and protein levels to the level of β-actin control protein.

In ovarian carcinoma patient cohorts, mean Ct values of duplicates normalized to reference genes were used for calculating differences in transcript levels between tissue types using the REST 2009 Software v1.0 (Qiagen), as published [72]. For relative gene expression, the 2^−ΔCt^ method and standard deviation was used [71]. Associations of transcripts with clinical data—age at diagnosis in years; histological type of ovarian carcinoma (serous vs. other); histological grade, G1 or G2 vs. G3 or G4; FIGO stage, I or II vs. III or IV and Ki-67 expression in %, progression of disease, death, and resistance to therapy—were assessed by the non-parametric Mann-Whitney, Kruskal–Wallis, and Spearman rank tests. Time to progression (TTP) was defined as the time elapsed between the surgical treatment and disease progression or cancer-related death. The survival functions were computed by the Kaplan–Meier method. Cut-offs defined by quartiles were tested and the “optimal cut-off” was defined as the highest statistical significance by the log-rank test. A *p*-value of <0.05 was considered statistically significant. All *p*-values are departures from two-sided tests. Statistical analyses were performed using SPSS v16.0 software (SPSS Inc., Chicago, IL, USA). Type I error in single gene expression analyses was controlled by the false discovery rate (FDR) test according to Benjamini and Hochberg [73] and adjusted *p*-values are provided for each comparison except for in vitro and in vivo analyses.

## Figures and Tables

**Table 1:** Clinical characteristics of EOC patients in the study.

Characteristics	EOC Set
	*n* (%) *
Mean age at diagnosis, years	59.8 ± 10.8
FIGO Stage	
I	8 (7.1)
II	11 (9.7)
III	83 (73.4)
IV	9 (8.0)
Not available	2 (1.8)
EOC type	
HGSC	90 (79.6)
Others	21 (18.6)
Not available	2 (1.8)
Histological grade	
G1	7 (6.2)
G2	18 (15.9)
G3	87 (77.0)
Not available	1 (0.9)
Progression	
Present	69 (61.0)
Absent	43 (38.1)
Not available	1 (0.9)
Death	
Present	43 (38.1)
Absent	70 (61.9)
Response	
Fully platinum-sensitive	70 (61.9)
Platinum–resistant	23 (20.4)
Partially platinum-sensitive	15 (13.3)
Not available	5 (4.4)
Time to progression	
Median ± SD (months)	22.0 ± 18.9
Number of evaluated patients	109 (96.5)
Treatment	
Pretreatment group	89 (78.8)
Posttreatment group	24 (21.2)
Therapeutic regimens	
*Adjuvant Therapy of Pretreatment group*	
Paclitaxel and platinum derivatives	80 (89.9)
Platinum derivatives	3 (3.4)
Unknown	6 (6.7)
*Posttreatment group*	
*Neoadjuvant Therapy of Posttreatment Group*	
Paclitaxel + platinum derivatives	23 (95.8)
Cisplatin + etoposide	1 (4.2)
*Adjuvant Therapy of Posttreatment Group*	
Paclitaxel + Platinum derivatives	21 (87.5)
Cisplatin + Etoposide	2 (8.3)
Platinum derivatives	1 (4.2)

Footnotes: * Number of patients with percentage in parentheses is shown. EOC = epithelial ovarian cancer, SD = standard deviation.

**Table 2:** Significant differences in the relative transcript levels of *TRIP6, CPS1,* and *ABCC3* mRNA between pretreatment (*n* = 89) and posttreatment (*n* = 24) ovarian carcinoma samples and control ovarian tissue samples (*n* = 17). Up = upregulation, down = downregulation, NS = not significant. *p*-value calculated by the REST2009 Software program (* *p* < 0.05, *** *p* < 0.001).

Gene	EOC Pretreated Tumors vs. Control Ovarian Tissue	EOC Posttreated Tumors vs. Control Ovarian Tissue
*ABCC3*	up *	NS
*CPS1*	down ***	down ***
*TRIP6*	down ***	down ***

## Data Availability

All the data are available upon reasonable request to the corresponding author.

## Supplementary Materials

The following are available online at https://www.mdpi.com/article/10.3390/ijms23010073/s1.
