# Supplementary material for: The Role of TRIP6, ABCC3 and CPS1 Expression in Resistance of Ovarian Cancer to Taxanes
Source: Int J Mol Sci. 2021 Dec 22;23(1):73. doi: 10.3390/ijms23010073 (PMC8744980; doi:10.3390/ijms23010073)

Supplementary materials

"The role of TRIP6, ABCC3 and CPS1 expression in resistance of ovarian cancer to taxanes"

Figure S1: Original western blot images for Figure 2

Part 2B

CPS1

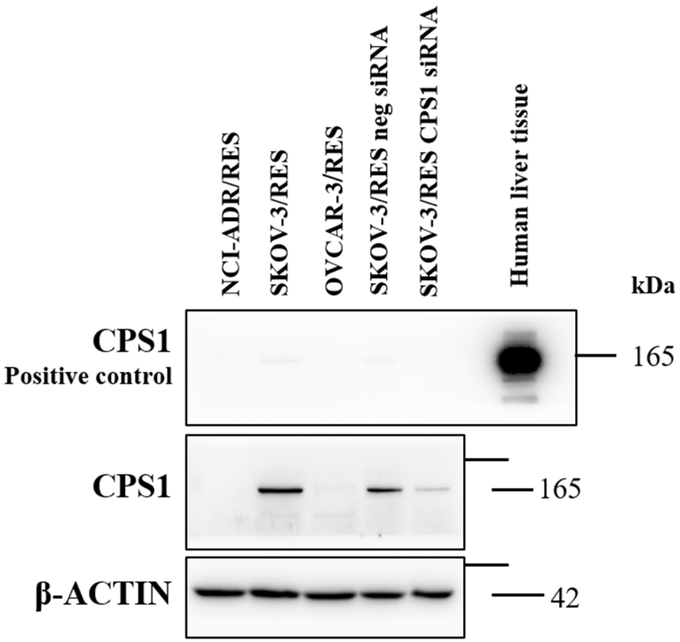

Part 2C

ABCC3

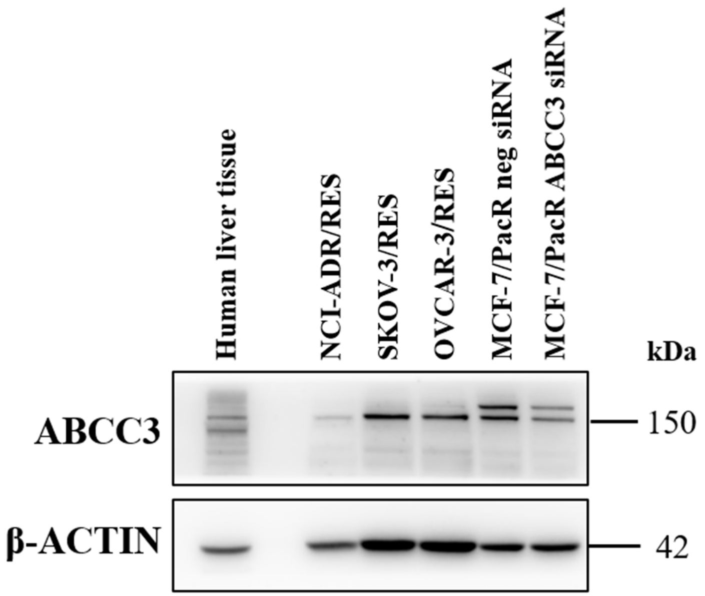

Part 2D – Samples marked with orange arrow were excluded from Figure 2D in the manuscript.

TRIP6

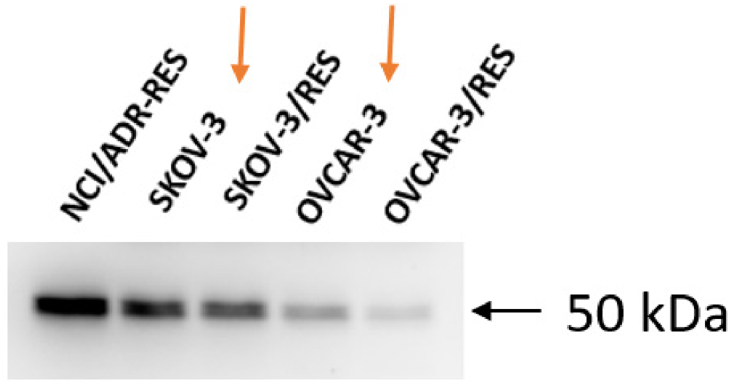

β-ACTIN

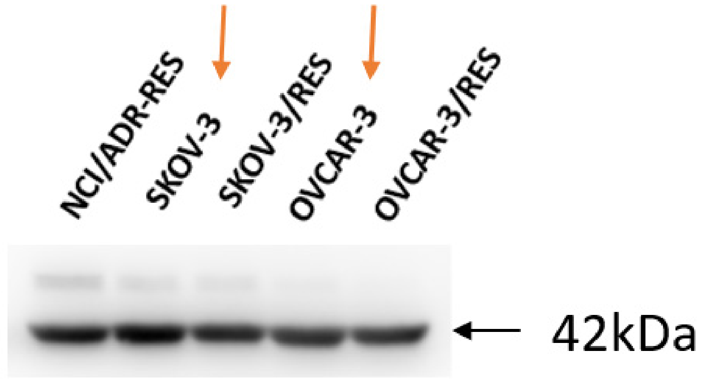

Figure S2: Original western blot images for Figure 3. Samples marked with blue arrow are included in Figure 3 in the manuscript.

CPS1

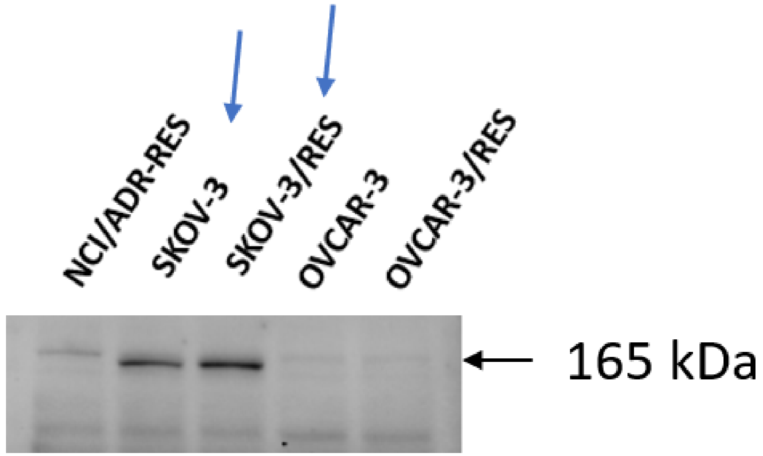

β-ACTIN

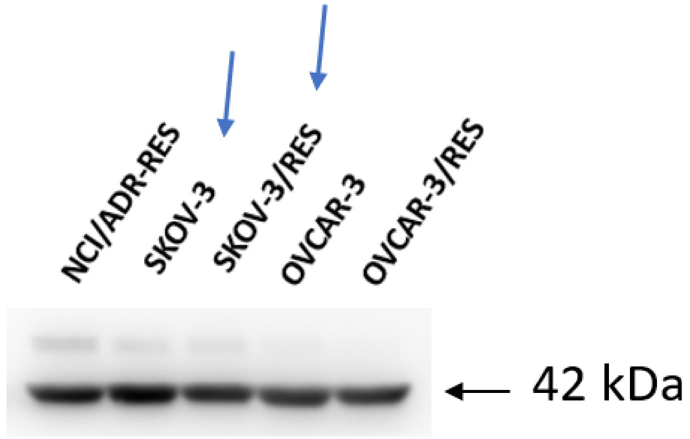

Supplement: Supplementary file 1 [file ijms-23-00073-s001.zip › ijms-1453107-supplementary.pdf]
